# Cannabis involvement in posttraumatic stress disorder emergency department visits after cannabis legalization

**DOI:** 10.1111/ajad.70014

**Published:** 2025-03-02

**Authors:** Laurent Perrault‐Sequeira, Michael Pugliese, Rachael MacDonald‐Spracklin, Jennifer Xiao, Stephen McCarthy, Daniel T. Myran

**Affiliations:** ^1^ Department of Internal Medicine University of Ottawa Ottawa Ontario Canada; ^2^ IC/ES uOttawa, Ottawa Hospital Research Institute Ottawa Ontario Canada; ^3^ Clinical Epidemiology Program Ottawa Hospital Research Institute Ottawa Ontario Canada; ^4^ Bruyère Health Research Institute Ottawa Ontario Canada; ^5^ Department of Family Medicine University of Ottawa Ottawa Ontario Canada

## Abstract

**Background and Objectives:**

Individuals with posttraumatic stress disorder (PTSD) have an elevated risk of cannabis use disorder. However, the effect of cannabis legalization on use among individuals with PTSD is unclear. We evaluated changes in cannabis involvement in emergency department (ED) visits for PTSD after medical and nonmedical legalization in Ontario, Canada.

**Methods:**

This repeated cross‐sectional study used health administrative data to identify all ED visits for PTSD among Ontario residents aged 10–105 between 2008 and 2022 (*n* = 15.7 million). We identified PTSD ED visits with a co‐diagnosis of cannabis (main exposure) or alcohol (control condition) and examined changes across four policy periods (medical legalization with restrictions, expanded medical legalization, nonmedical legalization with restrictions, and nonmedical commercial expansion) using Poisson models.

**Results:**

Among 381,450 PTSD ED visits, 4593 (1.29%) co‐involved cannabis and 11,625 (3.05%) co‐involved alcohol. Rates of cannabis involvement in PTSD ED visits increased by 151% (Incidence Rate Ratio [IRR]: 2.51; 95% CI: 2.24, 2.82) between the first and last policy periods (0.13 vs. 0.33 per 100,000 individuals), while alcohol‐involvement increased by 58% (IRR: 1.58; 95% CI: 1.47, 1.68). Cannabis involvement in PTSD ED visits increased steadily over the study period, with no significant association between policy periods and this trend.

**Discussion and Conclusions:**

Cannabis involvement in PTSD ED visits has increased over time during a period of liberalization of cannabis policy, but may be attributed to increased access and normalization rather than policy changes directly.

**Scientific Significance:**

Findings underscore the need for improved detection of and targeted interventions for disordered cannabis use among individuals with PTSD in regions with legalized cannabis.

## INTRODUCTION

Posttraumatic stress disorder (PTSD) is a psychiatric diagnosis with a significant healthcare burden characterized by exposure to a traumatic stressor, intrusive recollection, avoidance behaviors, negative mood and cognition states, and alterations in arousal and reactivity.^1^ PTSD affects approximately 8% of adults and is often accompanied by various comorbidities, including depressive and anxiety disorders.^2^, ^3^ There is increasing interest in the use of cannabis to manage symptoms of PTSD, especially as its cannabidiol (CBD) component helps to increase serotonin and dopamine levels.^4^ In Canada, more than 20% of those with PTSD experiencing moderate to severe symptoms reported using cannabis for medical reasons at least once in the past month.^5^, ^6^ However, there is limited and low‐quality evidence available on whether or not cannabis is effective for such relief. While some studies demonstrate symptom reductions and improvements in functional outcomes associated with the use of medical cannabis products, others suggest that cannabis use may worsen PTSD symptoms or lead to other adverse effects.^4^

In addition, individuals with PTSD are at elevated risk of substance use disorders, including cannabis use disorder (CUD).^7^ For example, a study on US veterans with co‐morbid mental health conditions found large increases in CUD over the past 15 years.^8^ Importantly, an increasing number of countries and states have legalized medical and nonmedical cannabis.^9^ One potential public health concern is that increasing access to cannabis may result in increases in CUD in individuals with PTSD. However, there is limited research examining how policy changes, such as medical and nonmedical cannabis legalization, may influence trends of cannabis use, CUD, and related harms in PTSD populations specifically, despite their increased vulnerability.

In Canada, access to cannabis has changed substantially over time, evolving from restricted medical access to broad legalization for both medical and nonmedical use. Medical cannabis access was first permitted in 2001 following the implementation of the Marihuana Medical Access Regulation, allowing individuals with a specific list of severe or chronic medical conditions to obtain approval from the Federal Government.^10^, ^11^ Access was expanded beyond this restrictive condition list in 2014, with the implementation of the Marihuana for Medical Purposes Regulations, allowing any individuals who received authorization from a physician to access medical cannabis for any condition that would benefit from its use.^11^, ^12^ The Federal Government announced its commitment to legalizing nonmedical cannabis in 2015, which then came into effect in October 2018.^13^, ^14^ For the first year and a half after legalization, there were restrictions on the number of cannabis stores and the types of legal cannabis products (flower, seeds, and oils only) allowed to be sold.^15^ In early 2020, high‐THC products (e.g., concentrates, vapes, and edibles) became newly available, and the provincial government lifted restrictions on the number of cannabis retail outlets, prompting a vast expansion in commercial access.^16^, ^17^, ^18^, ^19^ Despite these major policy shifts, the effects of cannabis legalization on disordered cannabis use among individuals with PTSD remains largely unexplored. To fill these gaps, the current study evaluated changes in cannabis involvement in ED visits for PTSD after medical and nonmedical legalization in the province of Ontario, Canada.

## METHODS

### Study design, population, and data sources

This population‐level repeated cross‐sectional study used the interrupted time series (ITS) method to assess trends in emergency department (ED) visits for PTSD and cannabis use/intoxication in the province of Ontario, Canada. We used linked health administrative database data held at ICES (formerly the Institute for Clinical Evaluative Sciences) to identify all ED visits for PTSD in Ontario between January 2008 and December 2022. ICES is an independent, non‐profit research institute whose legal status under Ontario's health information privacy law allows it to collect and analyze health care and demographic data without consent for health system evaluation and improvement.^20^

Included in our search were all ED visits from all individuals aged 10–105 years who were eligible for the province's public health coverage, which provides universal access to all hospital and medically necessary physician‐based services. Our cohort included 15.7 million people after excluding non‐Ontario residents and anyone not eligible for OHIP for a 2‐year period before study eligibility. Study data, which included baseline demographics and recent healthcare usage of individuals in captured PTSD ED visits, were pulled from six different databases, including the National Ambulatory Care Reporting System and the Registered Persons Database. These datasets were linked using unique encoded identifiers and analyzed at ICES. The use of the data in this project is authorized under section 45 of Ontario's Personal Health Information Protection Act and does not require review by a Research Ethics Board. This project was approved by the privacy office at ICES, and the STROBE reporting guidelines were followed.^21^

### Exposures

We divided our study into four time periods of distinct cannabis policy/legislation in Ontario. Period 1 (P1, January 1, 2008–November 30, 2015) coincides with a period when only medical cannabis was legally accessible, and more stringent restrictions were in place for obtaining a prescription. Period 2 (P2, December 1, 2015–September 30, 2018) coincides with the lifting of many of these restrictions, and greater accessibility to medical cannabis with a doctor's prescription. Period 3 (P3, October 1, 2018–February 29, 2020) coincides with the legalization of nonmedical cannabis in Canada on October 17, 2018, and significant restrictions on retail stores and types of cannabis products sold in Ontario. Period 4 (P4, March 1, 2020–December 31, 2022) coincides with the lifting of restrictions on retail stores and the rapid expansion of the commercial market in Ontario. Period 4 also coincides with the onset of the global COVID‐19 pandemic in March 2020.

### Outcomes

The primary outcome of the study was an incident ED visit due to PTSD with cannabis involvement. Among all eligible ED visits during the study period, visits for PTSD were identified when an International Classification of Diseases (ICD) 10th revision codes for PTSD (F43.x) were listed as the main or contributing reason for the ED visit based on the clinical judgment of the treating team. Among PTSD ED visits, we identified cannabis involvement when one of the following ICD‐10 codes was listed as the main or contributing reason for the visit based on the clinical judgment of the treating team.: T40.7 (Poisonings by cannabis, including derivatives) and F12.X (Mental and behavioral disorders due to use of cannabinoids). The control condition was PTSD ED visits with co‐involvement of alcohol, identified with ICD‐10 codes for a mental and behavioral disorder due to alcohol use (F10.x) or ethanol poisoning (T51.0) again as a main or contributing reason for the visit.

### Covariates

At the time of each index ED visit for PTSD, demographic details were obtained, including age, sex, rurality, and neighborhood income quintile. Rurality and neighborhood income quintile were obtained from the Postal Code Conversion File+ (PCFF+).^22^ We also identified outpatient visits (to a family physician or psychiatrist), ED visits, and hospitalizations for mental health and substance use/addictions visits during the 2 years preceding the index PTSD ED visit using validated diagnostic and billing codes (OHIP billings for outpatient visits, and ICD‐9 or ICD‐10 for ED visits or hospitalizations) from the Mental Health and Addictions Scorecard and Evaluation Framework indicator.^23^

### Statistical analyses

We calculated monthly ED visit rates per 100,000 individuals and per 1000 PTSD ED visits of our primary and control outcomes over the course of the study period (January 2008–December 2022). We used Quasi‐Poisson models to compare changes in the mean monthly rate of visits across each of the four policy periods using incidence rate ratios (IRRs) with 95% confidence intervals (CIs).

We then used an ITS design with segmented regression Poisson models to assess immediate and gradual changes in PTSD ED visit rates associated with cannabis legalization and commercialization. We examined changes in the monthly count of the various ED visit types, including the outcome and control conditions, offset by the log‐transformed relevant population (either the total population aged 10–105 at risk each month or the total number of PTSD ED visits each month). We included terms for a slope and level change for each policy interruption/intervention (the time point at which one policy period ends and the subsequent one begins) to examine the immediate and gradual effects of each policy. All analyses included first‐order autocorrelation and were adjusted for seasonality. Statistical significance was determined when the regression coefficient 95% CIs did not cross one. Statistical analyses were conducted using SAS Enterprise Guide version 7.1 (SAS Institute).

## RESULTS

Table 1 outlines the characteristics of individuals with an incident ED visit for PTSD. During the study period, there were 261,781 individuals with PTSD ED visits, of which 4377 (1.7%) had the involvement of cannabis, and 10,617 (4%) had the involvement of alcohol. Compared to PTSD ED visits without a substance involved, cannabis‐involved PTSD ED visits were more likely to be male (59.4% vs. 43.8%), younger (mean [SD] age of 26.22 [11.10] vs. 34.67 [17.61]) and have had a mental health acute care visit (49.6% vs. 31.6%) or substance use acute care visit (20.9% vs. 9.8%) in the past 3 years. Those with alcohol‐involved PTSD ED visits were more similar to non‐cannabis‐involved PTSD ED visits than to cannabis‐involved ED visits.

**TABLE 1:** Characteristics of individuals with an incident ED visit for PTSD between January 2008 and March 2019.

Variable	PTSD ED visit (no cannabis involvement) (*n* = 257,404)	PTSD ED visit with cannabis involvement (*n* = 4377)	PTSD ED visit with alcohol involvement (*n* = 10,617)	Standardized difference
Sex				
Female	144,769 (56.2%)	1778 (40.6%)	4955 (46.7%)	0.32
Male	112,635 (43.8%)	2599 (59.4%)	5662 (53.3%)	0.32
Age				
Mean age ± SD	34.67 ± 17.61	26.22 ± 11.10	37.79 ± 15.05	0.57
Age 10–18	54,607 (21.2%)	1285 (29.4%)	780 (7.3%)	0.19
Age 19–24	42,429 (16.5%)	1181 (27.0%)	1875 (17.7%)	0.26
Age 25–44	87,121 (33.8%)	1524 (34.8%)	4329 (40.8%)	0.02
Age 45+	73,247 (28.5%)	387 (8.8%)	3633 (34.2%)	0.52
Rurality				
Non‐specified rurality	1369 (0.5%)	22 (0.5%)	67 (0.6%)	0.00
Urban	217,720 (84.6%)	3948 (90.2%)	9156 (86.2%)	0.17
Rural	38,315 (14.9%)	407 (9.3%)	1394 (13.1%)	0.17
Neighborhood income quintile				
1 (Lowest/poorest)	74,127 (28.8%)	1307 (29.9%)	3336 (31.4%)	0.02
2	54,814 (21.3%)	944 (21.6%)	2226 (21.0%)	0.01
3	47,458 (18.4%)	770 (17.6%)	1856 (17.5%)	0.02
4	42,196 (16.4%)	680 (15.5%)	1638 (15.4%)	0.02
5 (Highest/richest)	36,741 (14.3%)	645 (14.7%)	1469 (13.8%)	0.01
Non‐specified	2068 (0.8%)	31 (0.7%)	92 (0.9%)	0.01
Mental health acute care visits in the past 3 years				
Any	81,375 (31.6%)	2172 (49.6%)	4466 (42.1%)	0.37
Mood disorder	37,626 (14.6%)	1040 (23.8%)	2283 (21.5%)	0.23
Anxiety	26,542 (10.3%)	625 (14.3%)	1254 (11.8%)	0.12
PTSD (within the last 2 years)	26,092 (10.1%)	1069 (24.4%)	2070 (19.5%)	0.38
OCD	644 (0.3%)	20 (0.5%)	27 (0.3%)	0.03
Schizophrenia	13,471 (5.2%)	479 (10.9%)	521 (4.9%)	0.21
Self‐harm	14,974 (5.8%)	478 (10.9%)	1290 (12.2%)	0.19
Other	15,471 (6.0%)	529 (12.1%)	802 (7.6%)	0.21
Substance use acute care visits in the past 3 years				
Any	25,281 (9.8%)	914 (20.9%)	3689 (34.7%)	0.31
Cannabis	2490 (1.0%)	289 (6.6%)	131 (1.2%)	0.30
Alcohol	13,276 (5.2%)	333 (7.6%)	3163 (29.8%)	0.10
Opioids	3150 (1.2%)	78 (1.8%)	170 (1.6%)	0.05
Other	11,141 (4.3%)	448 (10.2%)	1009 (9.5%)	0.23
Unspecified	2836 (1.1%)	118 (2.7%)	455 (4.3%)	0.12
Outpatient mental health or substance use visits in the past 3 years				
Any	172,008 (66.8%)	3409 (77.9%)	7701 (72.5%)	0.25
Family physician	159,170 (61.8%)	3106 (71.0%)	7176 (67.6%)	0.19
Psychiatrist	79,723 (31.0%)	1986 (45.4%)	3889 (36.6%)	0.30

Abbreviations: ED, emergency department; OCD, obsessive‐compulsive disorder; PTSD, posttraumatic stress disorder; SD, standard deviation.

The total and mean monthly rates of PTSD ED visits are outlined in Table 2. The mean monthly rates of non‐substance‐involved PTSD ED visits increased by 36% (IRR: 1.36; 95% CI: 1.29, 1.43) between P1 and P4 (14.06 vs. 19.21 per 100,000 individuals). Rates of cannabis involvement in PTSD ED visits increased by 151% (incidence rate ratio [IRR]: 2.51; 95% CI: 2.24, 2.82) between P1 and P4 (0.13 vs. 0.33 per 100,000 individuals), while those with alcohol involvement increased by 58% (IRR: 1.58; 95% CI: 1.47, 1.68). These trends were similarly observed using mean monthly rates per 1,000 PTSD ED visits; those with cannabis involvement increased by 85% (IRR: 1.85; 95% CI: 1.69, 2.03) between P1 and P4 (8.82 vs. 17.12 per 1,000 PTSD ED visits) while those with alcohol involvement increased by 16% (IRR: 1.16; 95% CI: 1.10, 1.23).

**TABLE 2:** Emergency Department visits for PTSD with and without cannabis and alcohol by cannabis legalization policy period.^a^

	Period 1	Period 2	Period 3		Period 4			
Characteristics	January 2008–November 2015	December 2015–September 2018	October 2018–February 2020		March 2020–December 2022	Period 2 versus Period 1	Period 3 versus Period 1	Period 4 versus Period 1
	**Total visits, No. (%)**							
Total PTSD ED visits	163,338	92,273	50,450	75,389				
Cannabis involvement	1506 (0.92)	1114 (1.21)	683 (1.35)	1290 (1.71)				
Alcohol involvement	4776 (2.92)	2781 (3.01)	1503 (2.98)	2565 (3.40)				
	**Mean monthly rate per 100,000 individuals**					**IRR (95% CI)**		
Total PTSD ED visits	14.06	20.83	22.24	19.21		1.48* (1.41–1.55)	1.58* (1.49–1.68)	1.36* (1.29–1.43)
Cannabis involvement	0.13	0.25	0.30	0.33		1.93* (1.71–2.18)	2.33* (2.02–2.68)	2.51* (2.24–2.82)
Alcohol involvement	0.41	0.63	0.66	0.65		1.52* (1.42–1.62)	**1.62** (1.50–1.76)	1.58* (1.47–1.68)
	**Mean monthly rate per 1000 PTSD ED visits**					**IRR (95% CI)**		
Cannabis involvement	8.82	12.02	13.58	17.12		1.31* (1.19–1.43)	1.47* (1.32–1.64)	1.85* (1.69–2.03)
Alcohol involvement	29.41	30.14	29.84	34.18		1.03 (0.97–1.08)	1.02 (0.96–1.09)	1.16* (1.10–1.23)

*Note*: Values of scientific significance are marked * and bolded. IRRs with 95% CIs that do not include 1 were considered statistically significant.

Abbreviations: CI, confidence interval; ED, emergency department; IRR, incidence rate ratio; PTSD, posttraumatic stress disorder.

^a^ P1 allowed access to medical cannabis for a limited number of conditions; P2 allowed access to medical cannabis for a wide number of medical conditions with physician authorization and the Federal Government announcement that nonmedical cannabis will be legalized (December 2015); P3 was the legalization of nonmedical cannabis with restrictions on the number of retail stores and types of products (flower only); P4 was the commercial expansion of nonmedical cannabis in Ontario with expansion on store numbers and product types which overlaps with the COVID‐19 pandemic.

**FIGURE 1:**
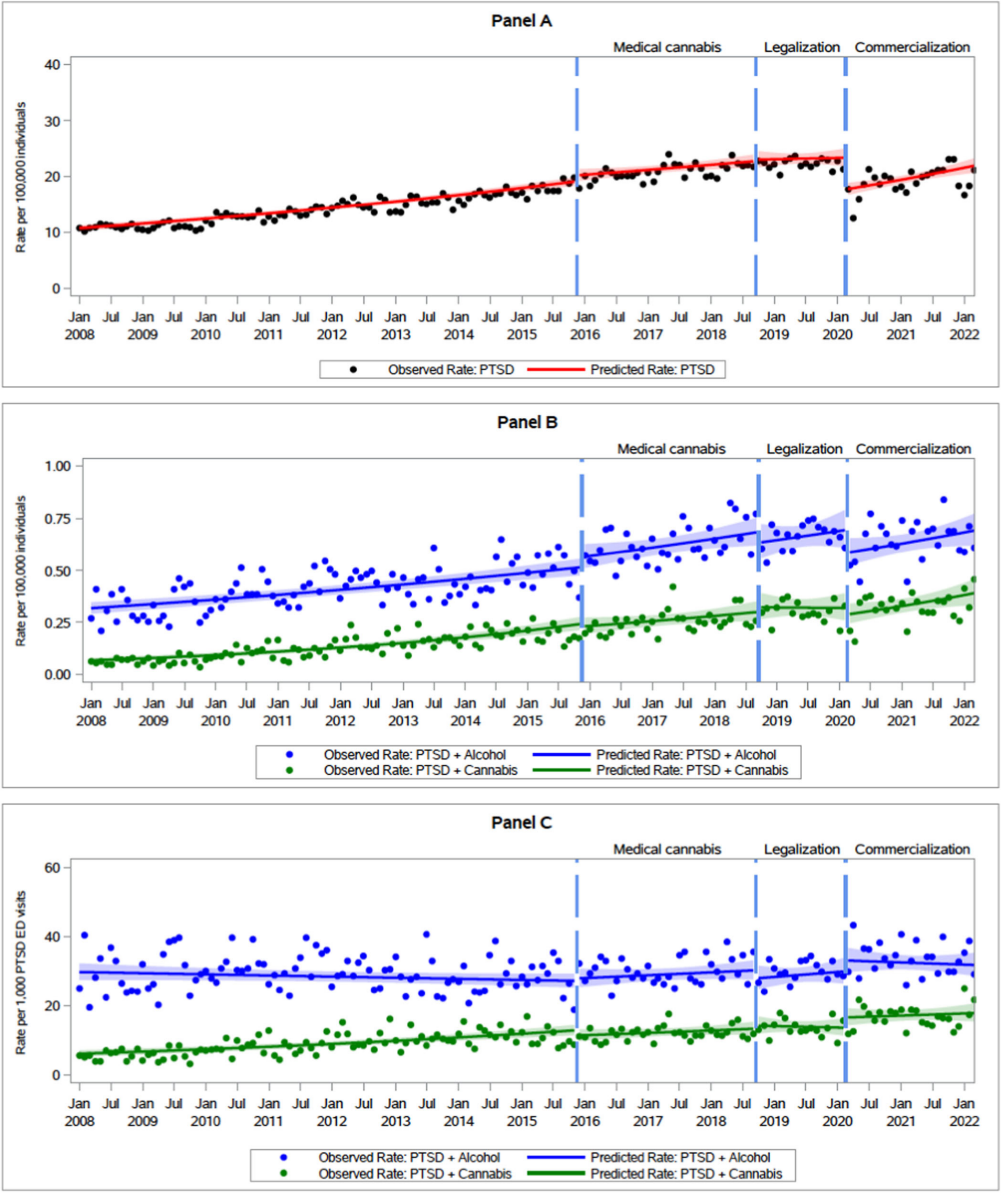
Trends in monthly emergency department (ED) visits for posttraumatic stress disorder (PTSD) and PTSD with co‐diagnosis of cannabis or alcohol use over time across different Canadian cannabis policy periods. Time series showing observed and predicted monthly rates of ED visits due to PTSD (A) per 100,000 individuals (B) with co‐involvement of cannabis (green) and with co‐involvement of alcohol (blue) (C) with co‐involvement of cannabis and with co‐involvement alcohol, per 1000 total PTSD ED visits. The vertical dashed lines denote four different policy periods of cannabis legalization in Canada.

Mean monthly ED visit rates over the four policy periods are shown in Figure 1. While PTSD ED visits with cannabis involvement increased steadily over the study period, no policy period was associated with a significant change in the underlying trend (see Table 3). For instance, in P1, the rate of PTSD ED visits with cannabis involvement per 100,000 people was increasing by 1.4% per month (IRR: 1.014; 95% CI: 1.011, 1.017). There was no significant slope or level changes during P2, P3, or P4, and rates of PTSD ED visits with cannabis involvement continued to increase by 1.2% (IRR: 1.012; 95% CI: 1.001, 1.023) during P4.

**TABLE 3:** Immediate and gradual changes in cannabis and alcohol involvement in PTSD ED visits during different policy periods.

	Incidence rate ratio (95% CI)				
Measure	PTSD ED visits per capita	PTSD + cannabis ED visits per capita	PTSD + alcohol ED visits per capita	PTSD + cannabis ED visits per 1000 PTSD ED visits	PTSD + alcohol ED visits per 1000 PTSD ED visits
**Period 1** a					
Pre‐slope	1.01* (1.01–1.01)	1.01* (1.01–1.02)	1.01* (1.00–1.01)	1.01* (1.01–1.01)	1.00 (1.00–1.00)
**Period 2** b					
Level change	1.06 (0.99–1.13)	0.95 (0.78–1.16)	1.10 (0.93–1.23)	0.88 (0.74–1.05)	1.02 (0.92–1.15)
Slope change	1.00 (1.00–1.00)	0.99 (0.99–1.00)	1.00 (1.00–1.01)	1.00 (0.99–1.00)	1.00 (1.00–1.01)
Overall slope	1.00* (1.00–1.01)	1.01 (1.00–1.02)	1.01* (1.00–1.01)	1.01 (1.00–1.01)	1.00 (1.00–1.01)
**Period 3** c					
Level change	1.01 (0.94–1.09)	1.08 (0.84–1.38)	0.93 (0.79–1.09)	1.07 (0.86–1.33)	0.92 (0.79–1.07)
Slope change	1.00 (0.99–1.00)	0.99 (0.97–1.01)	1.00 (0.99–1.01)	0.99 (0.97–1.01)	1.00 (0.99–1.01)
Overall slope	1.00 (0.99–1.01)	1.00 (0.98–1.02)	1.01 (0.99–1.02)	1.00 (0.98–1.01)	1.00 (0.99–1.02)
**Period 4** d					
Level change	0.75 (0.70–0.82)	0.90 (0.70–1.15)	0.84 (0.71–0.99)	1.21 (0.97–1.52)	1.11 (0.95–1.30)
Slope change	1.01 (1.00–1.02)	1.01 (0.99–1.04)	1.00 (0.99–1.02)	1.01 (0.99–1.03)	0.99 (0.98–1.01)
Overall slope	1.01* (1.00–1.01)	1.01* (1.00–1.02)	1.01 (1.00–1.01)	1.00 (0.99–1.01)	1.00 (0.99–1.01)

*Note*: Values of scientific significance are marked * and bolded. IRRs with 95%CIs that do not include 1 were considered statistically significant.

Abbreviations: ED, emergency department; PTSD, posttraumatic stress disorder.

^a^ P1 allowed access to medical cannabis for a limited number of conditions.

^b^ P2 allowed access to medical cannabis for a wide number of medical conditions with physician authorization and the Federal Government announcement that nonmedical cannabis will be legalized (December 2015).

^c^ P3 was the legalization of nonmedical cannabis with restrictions on the number of retail stores and types of products (flower only).

^d^ P4 was the commercial expansion of nonmedical cannabis in Ontario with an expansion on store numbers and product types, which overlaps with the COVID‐19 pandemic.

There was a significant immediate 24.6% (IRR: 0.754; 95% CI: 0.695, 0.820) decrease in rates of non‐substance‐involved PTSD ED visits per 100,000 individuals at the start of P4 (coinciding with the onset of the COVID‐19 pandemic) followed by a significant monthly slope increase (IRR: 1.008 95% CI: 1.000, 1.016) as rates of non‐substance involved PTSD ED visits gradually returned to baseline. At the start of P4, there were smaller and nonsignificant immediate decreases in per capita rates of cannabis‐involved and alcohol‐involved PTSD ED visits. When examining the immediate changes in cannabis and alcohol involvement per 1000 PTSD ED visits, there was an insignificant 21.5% increase (IRR: 1.215; 95% CI: 0.973, 1.51) in those with cannabis involvement and an insignificant 10.8% (IRR: 1.108; 95% CI: 0.948, 1.30) increase in those with alcohol involvement at the start of P4.

## DISCUSSION

This repeated cross‐sectional study found that the proportion of PTSD ED visits with cannabis involvement in Ontario, Canada, almost doubled between the pre‐legalization and post‐legalization/commercialization periods. Over the study period, increases in PTSD ED visits with cannabis involvement were greater than in those with alcohol involvement (151% vs. 58%). The increase in visits with cannabis involvement was generally steady throughout the study period, and we did not observe significant changes to this trend during time periods that coincided with significant changes to cannabis policy and legislation (most notably the legalization of nonmedical cannabis in Canada in October 2018, and the significant expansion in commercial access to cannabis products starting in March 2020 following lifting of restrictions on retail stores and types of cannabis products). The observed increases over time in the involvement of cannabis use in PTSD ED visits are consistent with increases in cannabis use and CUD in Ontario over the past 15 years.^24^, ^25^, ^26^ These finding suggests that cannabis involvement among those with PTSD may be influenced more by the growing normalization and social acceptance of cannabis use than by specific regulatory or policy changes. Furthermore, it may indicate that nonmedical cannabis legalization and commercialization serve as part of a larger social trend, where increased product availability, decreased stigma, and the presence of medical claims may encourage self‐medication practices among those living with PTSD.

Many studies show that some individuals with PTSD use cannabis for symptom relief.^27^, ^28^ Over 12% of medical cannabis users in Canada report PTSD as their primary condition for medication, and 21% of Canadians with PTSD indicated they used cannabis in the past 30 days for symptom relief.^5^, ^6^, ^29^ However, it remains unclear whether individuals with PTSD are increasingly using cannabis to alleviate their symptoms. While there may be potential for cannabis and synthetic cannabinoids to 1 day have a role in the treatment of PTSD, high quality evidence to support its routine use in PTSD is lacking.^30^, ^31^ This knowledge gap is particularly important, as self‐medicating with cannabis may risk exacerbating PTSD symptoms or contributing to dependency.

Prior work has found a positive association between PTSD symptom severity and problematic cannabis use, indicating that individuals with more severe PTSD symptoms are more likely to engage in more frequent cannabis use.^32^ This bidirectional relationship suggests that PTSD symptoms may drive individuals toward higher cannabis use as a coping mechanism, which could unintentionally worsen symptoms over time. Our findings indicate that the significant increase in rates of PTSD ED visits with cannabis involvement may, in part, reflect this bidirectional relationship where more severe PTSD symptoms lead to increased cannabis use, which may, in turn, worsen PTSD symptoms. Overall, this relationship is concerning given that PTSD with a co‐occurring substance use disorder presents substantial clinical challenges, including being more difficult to treat, poorer health outcomes, and higher rates of suicide attempts.^33^

Our findings also highlight that a substantial proportion of PTSD ED visits with cannabis involvement occurred among youth aged 24 or younger, representing over 56% of cases compared to just under 38% of cases without cannabis involvement. Males (59%) were also more likely than females (49%) to have a PTSD ED visit with cannabis involvement. Several mechanisms may be driving these findings, including higher baseline rates of substance use in men and younger adults.^34^ The literature also highlights sex and gender differences in coping strategies, with males or men being more likely to engage in externalizing behaviors such as substance use in response to trauma.^35^, ^36^ Moreover, adolescents with PTSD may be particularly vulnerable to using cannabis as a means of coping with distressing symptoms, given that PTSD during this developmental period is associated with heightened emotional sensitivity, reactivity, and impulsivity.^37^, ^38^ Younger age of cannabis use initiation is associated with a higher likelihood of frequent cannabis use and the development of a CUD later in life,^39^, ^40^ and, as such, may contribute to a cycle of increased use and worsening PTSD symptom severity over time.

Our findings further underscore the importance of EDs as critical points for screening and early intervention for CUDs among individuals with PTSD. ED visits provide an opportunity to connect patients with specialized addiction services and mental health and psychiatric resources, allowing for comprehensive screening for CUDs and, where possible, linking patients with integrated care pathways. Our findings indicating the disproportionate representation of males and youth in PTSD ED visits involving cannabis warrant the prioritization of targeted prevention and intervention strategies, and may merit additional screening for disordered cannabis use in regions with legal cannabis for these groups. Proactive screening coupled with greater awareness of increases in cannabis involvement in ED visits among those with PTSD may not only help identify cannabis‐related risks in this population but also support more effective and comprehensive treatment plans that address both PTSD and substance use needs.

### Limitations

The findings of this study should be considered alongside some limitations. First, our study was limited by the lack of detail on patterns of cannabis use, whether or not individuals were self‐medicating PTSD symptoms with cannabis, or if they had a diagnosed CUD. Without information on the motivations or patterns of cannabis use, it is challenging to discern whether increased ED visits with cannabis involvement is primarily driven by self‐medication practices or other factors. Similarly, by focusing only on high‐risk cannabis use and PTSD, the study may underestimate overall cannabis use trends among individuals with PTSD. This narrower scope could obscure patterns in less harmful use levels, limiting the study's insights into broader cannabis use behaviors in this population. In addition, while we observed no association between specific policy changes and cannabis involvement in PTSD ED visits, our study design faces challenges differentiating the impact of policy changes from changing social norms and behaviors in the lead‐up to legalization. The effects of the COVID‐19 pandemic and its impacts on both ED use patterns and cannabis use in patients with PTSD should be considered when interpreting our findings. Finally, the legal cannabis retail market in Canada continues to expand. Evidence suggests that greater access to high‐potency cannabis products and cannabis retail stores is associated with increased cannabis use and harms, and ongoing monitoring of cannabis involvement in PTSD once the market reaches maturity is indicated.^41^, ^42^, ^43^

## CONCLUSION

Our findings demonstrate that ED visits related to PTSD with the involvement of cannabis use increased substantially during a period of increasing medical and nonmedical use of cannabis. Cannabis involvement in PTSD ED visits increased steadily over time and did not accelerate following medical or nonmedical cannabis legalization or commercialization. Greater efforts by clinicians to identify cannabis use in individuals with PTSD and offer appropriate counseling and treatment may be indicated.

## CONFLICT OF INTEREST STATEMENT

The authors declare no conflicts of interest.

## Data Availability

The data set from this study is held securely in coded form at ICES. Although data sharing agreements prohibit ICES from making the data set publicly available, access may be granted to those who meet pre‐specified criteria for confidential access, available at www.ices.on.ca/DAS. The full data set creation plan and underlying analytic code are available from the authors on request, understanding that the computer programs may rely upon coding templates or macros unique to ICES and are, therefore, inaccessible or may require modification.
